# Estimation of the Spontaneous Mutation Rate in *Heliconius melpomene*

**DOI:** 10.1093/molbev/msu302

**Published:** 2014-11-03

**Authors:** Peter D. Keightley, Ana Pinharanda, Rob W. Ness, Fraser Simpson, Kanchon K. Dasmahapatra, James Mallet, John W. Davey, Chris D. Jiggins

**Affiliations:** ^1^Institute of Evolutionary Biology, School of Biological Sciences, University of Edinburgh, Edinburgh, United Kingdom; ^2^Department of Zoology, University of Cambridge, Cambridge, United Kingdom; ^3^Department of Genetics, Evolution and Environment, University College London, London, United Kingdom; ^4^Department of Biology, University of York, York, United Kingdom; ^5^Department of Organismic and Evolutionary Biology, Harvard University

**Keywords:** mutation, *Heliconius*, genome sequencing

## Abstract

We estimated the spontaneous mutation rate in *Heliconius melpomene* by genome sequencing of a pair of parents and 30 of their offspring, based on the ratio of number of de novo heterozygotes to the number of callable site-individuals. We detected nine new mutations, each one affecting a single site in a single offspring. This yields an estimated mutation rate of 2.9 × 10^−9^ (95% confidence interval, 1.3 × 10^−9^–5.5 × 10^−9^), which is similar to recent estimates in *Drosophila melanogaster*, the only other insect species in which the mutation rate has been directly estimated. We infer that recent effective population size of *H. melpomene* is about 2 million, a substantially lower value than its census size, suggesting a role for natural selection reducing diversity. We estimate that *H. melpomene* diverged from its Müllerian comimic *H. erato* about 6 Ma, a somewhat later date than estimates based on a local molecular clock.

Understanding the process of spontaneous mutation is central for many of the most important questions in evolutionary genetics. The neutral nucleotide diversity expected within a species (θ) is proportional to the product of the spontaneous mutation rate per nucleotide site (*μ*) and the effective population size (*N*_e_). Variation in the mutation rate is therefore expected to contribute to the large range of variation in neutral nucleotide diversity that has been observed in natural populations (Leffler et al. 2012). Conversely, if nucleotide diversity for putatively neutral sites of a population has been estimated, and the mutation rate is known, it is possible to estimate *N*_e_. Effective population size is an important factor determining the effectiveness of natural selection, and selection effects on diversity at linked sites could limit diversity in the genome (Charlesworth B and Charlesworth D 2010). Species split times can be estimated using between-species neutral nucleotide divergence, which is also expected to be proportional to the mutation rate. This can be useful if fossil evidence-based dates of species divergence are not available. Estimates of the mutation rate for a range of species across the tree of life are therefore needed in order to better understand patterns of diversity in relation to *N*_e_ and the influence of natural selection on variation. However, at present, only a handful of direct mutation rate estimates are available, for a small number of model species.

Mutation rate estimation has until recently depended on assaying rates of mutation at specific loci or on the between-species nucleotide divergence at putatively neutral sites, such as synonymous sites (Drake et al. 1998). There are, however, drawbacks to these approaches, including uncertainty about species divergence dates and nonneutral synonymous site evolution (Chamary et al. 2006). This has led to efforts to directly estimate the mutation rate by sequencing mutation accumulation (MA) lines or outbred parents and their offspring. The MA line approach suffers from potential difficulties, however. For example, recessive mutator alleles might become fixed by inbreeding in the MA line progenitor. Furthermore, its practical applicability is limited, because inbred lines cannot be produced for most species. Sequencing parents and their offspring and searching for de novo mutations in the offspring are more generally applicable, but to date experiments have only been carried out in humans (Roach et al. 2010; Conrad et al. 2011; Kong et al. 2012; Michaelson et al. 2012) and *Drosophila melanogaster* (Keightley et al. 2014).

Both humans and *D. melanogaster* have “finished” genome sequences, but the genomes of most sequenced species are incomplete or “draft” and it is unknown whether parent–offspring sequencing can be applied in such cases. Widely used sequencing technologies produce short reads and spurious base calls arise due to the mismapping of paralogs. Here, we apply parent–offspring genome sequencing to a tropical butterfly species, *Heliconius melpomene*, whose genome sequence is currently draft (Heliconius Genome Consortium 2012). *Heliconius melpomene* has become a focal organism for genome-based studies of speciation and hybridization (Martin et al. 2013), and an accurate estimate of the mutation rate will have several immediate applications. We use deep sequencing of parents and 30 offspring to produce an estimate of the mutation rate that is close to one recently obtained by similar means in *D. melanogaster* (Keightley et al. 2014).

## Results

Among the 30 offspring, we sequenced 13 focal offspring at a high depth (mean = 26.3, SD = 7.1; supplementary table S1, Supplementary Material online) and 17 “bait offspring” at a lower depth (mean = 12.7, SD = 3.9). Bait offspring were used to remove regions prone to alignment errors that generate false positives by excluding sites at which any of these individuals had an alternate base call. Mutations were not called in the bait offspring nor did they contribute to the number of site-individuals. In 4,309 scaffolds of the draft genome assembly, there are 2.70 × 10^8^ sites, of which 1.23 × 10^8^ (46%) were estimated to be callable, yielding 1.60 × 10^9^ site-individuals.

We used the Genome Analysis Toolkit (GATK; Depristo et al. 2011) to call mutations. We assume that reads having the alternate allele at a site present in multiple individuals are due to mismapping. This arises when a paralogous locus present in the sample, but not in the reference genome, contributes reads that map to the wrong place (Li 2011). We assume that such mismapping is equally likely to occur at mutated and unmutated sites.

We applied the mutation calling rules described in Materials and Methods to the GATK genotype calls, yielding 15 candidate mutations appearing as de novo heterozygotes in up to two focal offspring (supplementary table S2, Supplementary Material online). We first examined each candidate using the Integrative Genomics Viewer (Thorvaldsdóttir et al. 2012) to determine whether there are single nucleotide polymorphisms (SNPs) in complete association with the alternate base calls at the candidate sites, a characteristic of mismapped paralogs (Li 2011; Keightley et al. 2014). Two closely linked candidates 17 bp apart on contig HE668478 (individual 110; supplementary fig. S1, Supplementary Material online) and candidates HE671028 and HE669561 (individual 110; supplementary figs. S2 and S3, Supplementary Material online) met this criterion. Furthermore, in each case reads containing the alternate allele are truncated and have unmapped mate pairs. We judged these four candidates as likely false positives caused by mismapping. We then attempted to verify the 11 remaining candidates by Sanger sequencing. Ten gave clearly interpretable chromatograms, confirming that eight are genuine mutations, and that candidates HE671439 and HE672001 are false positives (supplementary table S1, Supplementary Material online). Further attempts at sequencing the remaining candidate (HE671010) were unsuccessful, but mutant-bearing reads are well aligned and have aligned mate pairs (supplementary fig. S4, Supplementary Material online), suggesting that it is genuine. Thus, there are nine apparently genuine de novo mutations, eight confirmed by Sanger sequencing (table 1), each one affecting a single site and present in a single focal individual.

**Table 1.** Mutations Called and Depth of Sequencing Coverage Statistics for the Wild Type (WT) and Mutant (Mut) Bases in the Mutant Focal Offspring Along with Average Read Depth in the Parents and Focal Offspring.

Contig	Position	Individual	Base Call		Depth		Mean Depth	
			WT	Mut	WT	Mut	Parents	Offspring
HE671270	80778	118	A	T	5	15	33.5	26.8
HE670334	71590	33	T	C	13	11	33	28.7
HE670118	16036	103	G	A	11	14	17	25.0
HE670855	10858	37	A	G	25	11	30.5	32.7
HE668834	189330	1	G	A	26	23	58	35.6
HE671384	187868	4	T	A	29	17	40.5	30.8
HE672075	836004	118	G	A	4	12	17	15.6
HE679870	4463	33	G	C	24	13	53	34.2
HE671010	9591	4	T	A	21	21	24.5	22.0

Among the nine de novo mutations, the number of transitions exceeds the number of transversions, as is usually observed in eukaryotes. The number of mutations divided by twice the number of callable site-individuals yields an estimated mutation rate (uncorrected for false negatives) of 2.8 × 10^−^^9^ (95% confidence interval = 1.3 × 10^−^^9^–5.3 × 10^−^^9^, assuming that the number of mutations is Poisson distributed).

**Fig. 1.**
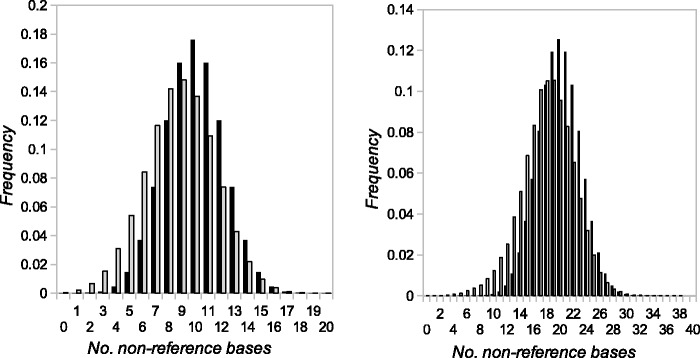
Examples of observed frequency distributions (red or gray) and binomial distributions with parameter 0.5 (blue or black) of alternate (i.e., nonreference) base number at heterozygous sites in the focal offspring having (*A*) 20 reads and (*B*) 40 reads.

To estimate the frequency of false negatives, we simulated synthetic mutations by modifying sequencing reads for randomly selected sites in the focal offspring. We realigned and analyzed the modified data using the same procedures as for the real data. Among 1,000 synthetic mutations, 456 occurred at callable sites where all other focal offspring, both parents and all bait offspring were pure. Of the callable mutations, 436 (96%) were called. The small proportion of uncalled mutations presumably reflects mutant-bearing reads mapping less frequently than reference reads (fig. 1). A corrected estimate for the mutation rate is therefore 2.9 × 10^−^^9^ (95% confidence interval = 1.3 × 10^−^^9^–5.5 × 10^−^^9^).

## Discussion

We estimated the mutation rate per base pair by genome sequencing of parents and offspring in *H. melpomene*. The incomplete state of the genome causes difficulties in identification of de novo mutations, because paralogous reads map more frequently to the wrong location, often yielding false heterozygote calls. Disregarding impure sites affecting any bait offspring and more than two focal offspring effectively addressed this problem. It has been estimated that approximately 20% of spontaneous recessive sex-linked lethal mutations in *D. melanogaster* males occur as premeiotic clusters (Woodruff and Thompson 1992). In the present experiment, we detected no mutation clusters affecting two focal offspring, but in view of the small number of mutation events detected, the sequences of many more individuals will be needed to accurately estimate the rate of premeiotic cluster mutations in *H. melpomene*. The draft state of the genome precluded the detection of large-scale de novo variants, such as rearrangements and duplications, which are particularly sensitive to mismapping.

Autosomal nucleotide diversity (*π*) at 4-fold degenerate sites in *H. melpomene* is approximately 2.4% (Martin SH, unpublished data). Assuming neutral synonymous sites evolution, and equating *π* to 4*N*_e_*μ*, *N*_e_ for the species is therefore approximately 2 million. This will be an underestimate if selection reduces diversity at 4-fold sites. However, estimates of *N*_e_ for *D. melanogaster* based on this approach are of similar magnitude (Keightley et al. 2014), but they are orders of magnitude smaller than both species’ census population sizes. Similar diversities and effective population sizes are consistent with the small range of genetic diversity across eukaryotes (Leffler et al. 2012), suggesting a role for processes such as genetic draft limiting diversity (Maynard-Smith and Haigh 1974; Gillespie 2001; Leffler et al. 2012).

Estimates of *μ* can also be used to date species divergences, assuming that neutral nucleotide divergence *d* = 2*μt*, where *t* is the divergence time in generations. For example, synonymous divergence between *H. melpomene* and its Müllerian comimic *H. erato* corrected for diversity within *H. melpomene* is 14% (Martin SH, unpublished data), yielding *t* = 23 million generations, which will be an underestimate if selection reduces synonymous site divergence. Assuming four generations per year, the divergence date is approximately 6 Ma, which is somewhat more recent than that estimated from a fossil-calibrated phylogeny of 10–13 Ma (Kozak et al. 2014). Although our data suggest that current estimates of the age of the *Heliconius* radiation are approximately correct, further work will be required to reconcile these estimates. It remains to be seen whether the hypothesis that the early radiation of *Heliconius* coincided with a time of rapid uplift in the Andes about 10 Ma is supported.

This is only the second direct estimate of the mutation rate per base pair in insects, and the first in Lepidoptera. There have been several direct estimates in *D. melanogaster,* by Denaturing High Performance Liquid Chromatography (Haag-Liautard et al. 2007), by whole-genome sequencing of MA lines (Keightley et al. 2009; Schrider et al. 2013), and most recently by parent–offspring sequencing (Keightley et al. 2014). There is significant variation among these estimates, but most are close to 3 × 10^−^^9^, which is remarkably close to our estimate of 2.9 × 10^−^^9^ for *Heliconius*. We have demonstrated that it is possible to estimate the mutation rate by offspring–parent genome sequencing for the case of a draft genome sequence. It should soon be possible to address the question of whether this lack of variation in the mutation rate extends to other arthropod groups whose draft genome sequences are now becoming available.

## Materials and Methods

### Cross Sequencing

The cross was previously used to produce chromosomal scaffolds of the *H. melpomene* genome (Heliconius Genome Consortium 2012, supplementary material section S4). After four generations of inbreeding, a male *H. melpomene melpomene* from the same lineage as for the *H. melpomene* reference genome was crossed with a female *H. melpomene rosina* from a laboratory strain established from Gamboa, Panama. DNA from two F_1_ parents and 30 of their F_2_ offspring was extracted using the DNeasy Blood and Tissue Kit (Qiagen). Illumina TruSeq libraries (300 bp insert size) were sequenced using 100-bp paired-end reads on an Illumina HiSeq2500.

### Alignment to Reference Genome

Reads for parents and offspring were aligned to version 1.1 of the *H. melpomene* genome (available on Ensembl and from http://butterflygenome.org, last accessed November 5, 2014) using SMALT version 0.7.0.1 with default options. Output sequence alignment/map (SAM) files were converted to binary format (BAM) files, sorted and annotated with read groups using Picard version 1.84 (http://picard.sourceforge.net/, last accessed November 5, 2014).

### Genotype Assignment

Each individual’s BAM file was processed to remove duplicates using Picard tools, then to realign indels using GATK. SNPs were called using the GATK UnifiedGenotyper across all individuals simultaneously to produce a variant call format (VCF) file, assuming a heterozygosity parameter of 0.01. For high read depth, genotype calls are insensitive to this parameter (Depristo et al. 2011; Ness et al. 2012).

### Mutation Calling

We processed the VCF by a similar algorithm as described previously (Keightley et al. 2014) filtering sites as follows:

1. Not marked as low quality (GATK LowQual).
2. Read depth of both parents ≥10, both homozygous references, containing no alternate allele reads.
3. None of the 17 bait offspring contains alternate allele reads.
4. The genotypes of all 13 focal offspring are defined (i.e., are called by GATK).
5. At most two focal offspring are called as heterozygous by GATK.
6. No other focal offspring contains alternate allele reads.

Our method excludes sites containing alternate alleles in either parent, which precludes the identification of mutations at polymorphic sites. Assuming that mutations are not more frequent at polymorphic sites, this should reduce the number of mutations and callable sites proportionally. There was no filtering carried out on read depth of the focal or bait offspring. Heterozygotes called among the focal offspring were marked as candidate mutations.

### Synthetic Mutations

We estimated the proportion of false negatives (genuine mutations we failed to call) by simulating mutations in the *Heliconius* data, running our pipeline, and calculating the fraction of simulated mutations called. Synthetic mutations were simulated by modifying the reads overlapping a random site in a focal offspring. We sampled the number of reads to be altered from empirical distributions of numbers of nonreference base calls at heterozygous sites (see below). For each synthetic mutation, we randomly sampled a genomic position *b* and a focal offspring. We sampled a random integer *y* from the frequency distribution of nonreference base number for the individual’s read depth (e.g., see fig. 1). We then changed *y* reference bases to a different randomly selected base by modifying reads overlapping position *b* in the individual’s BAM file.

We generated 1,000 synthetic mutations in the BAM files of focal individuals, extracted all reads from the modified BAM files, and aligned the modified reads to the reference genome by the procedure used for the original data. We then applied the mutation-calling algorithm, exactly as described above, with the exception that filters were not applied to the focal offspring carrying the synthetic mutation. The fraction of callable simulated mutated sites estimates the fraction of callable sites in the genome. Uncallable sites will include, for example, sites of low mapping quality and sites where genotypes are undefined in one or more focal offspring.

### Frequency Distributions of Nonreference Read Number in Heterozygotes

To produce the distributions used to generate synthetic mutations, we identified a set of sites heterozygous for natural polymorphisms, regardless of the genotypes called from the sequencing data, taking advantage of the lack of recombination in *Heliconius* females. F_2_ offspring receive whole chromosomes from the F_1_ mother, so SNPs from the same chromosome have identical segregation patterns in the offspring, and segregation patterns for each of the 21 *H. melpomene* chromosomes for this cross are known (Heliconius Genome Consortium 2012). We identified SNPs from each chromosome by compiling sites called as heterozygous in the F1 mother, homozygous in the F1 father, and matching one of the chromosome segregation patterns for the bait offspring. Heterozygous focal offspring could then be identified based on segregation pattern, without reference to their sequenced genotype. We used these heterozygous focal offspring to tabulate frequency distributions of numbers of nonreference base calls for read depths 1, … 100 (see fig. 1).

### Sanger Sequencing

With the exception of four candidates ruled out by inspection (see Results), we checked all candidates by Sanger sequencing. We sequenced the focal individual and one control individual on both strands. If the initial sequencing failed, an alternative primer pair was tried.

## Data Accessibility

Whole-genome sequence data for the parents and the 30 offspring from the mapping cross used for this study are available from the European Nucleotide Archive, study accession PRJEB7581.

## Supplementary Material

Supplementary figures S1–S4 and tables S1 and S2 are available at *Molecular Biology and Evolution* online (http://www.mbe.oxfordjournals.org/).

Supplementary Data
