# Supplementary material for: Estimation of the Spontaneous Mutation Rate in Heliconius melpomene
Source: Mol Biol Evol. 2014 Nov 3;32(1):239–43. doi: 10.1093/molbev/msu302 (PMC4271535; doi:10.1093/molbev/msu302)
Supplement: Supplementary Data [file supp_32_1_239__index.html]

Estimation of the Spontaneous Mutation Rate in Heliconius melpomene — Estimation of the Spontaneous Mutation Rate in Heliconius melpomene — Supplementary Data 

# Estimation of the Spontaneous Mutation Rate in *Heliconius melpomene*

## Supplementary Data

files

**Files in this Data Supplement:**

- Supplementary Data - pdf file
