# Supplementary material for: Estimation of the Spontaneous Mutation Rate in Heliconius melpomene
Source: Mol Biol Evol. 2014 Nov 3;32(1):239–43. doi: 10.1093/molbev/msu302 (PMC4271535; doi:10.1093/molbev/msu302)

SUPLEMENTARY MATERIAL

Estimation of the spontaneous mutation rate in *Heliconius melpomene*

Peter D. Keightley, Ana Pinharanda, Rob W. Ness, Fraser Simpson, Kanchon K. Dasmahapatra, James Mallet, John W. Davey, Chris D. Jiggins

Table S1. Average sequencing depths after removal of duplicate reads in the parents and focal offspring.

**Individual**	**Mean Depth**
Female parent	32.8
Male parent	27.8
Offspring 1	34.7
Offspring 111	21.6
Offspring 120	21.5
Offspring 33	22.5
Offspring 74	28.3
Offspring 103	26.9
Offspring 114	18.4
Offspring 2	33.0
Offspring 37	28.4
Offspring 110	23.4
Offspring 118	21.3
Offspring 31	18.7
Offspring 4	42.8

Table S2. Candidate mutations analysed.

			Base Call		*Depth*		*Mean Depth*			
Contig	*Position*	*Indiv*	*WT*	*Mut*	*WT*	*Mut*	*Parents*	*Offspring*	*Sanger Result*	*Mutation called?*
HE672001	2467	74	G	A	20	12	30	25.1	Negative	No
HE671270	80778	118	A	T	5	15	33.5	26.8	Positive	Yes
HE670334	71590	33	T	C	13	11	33	28.7	Positive	Yes
HE670118	16036	103	G	A	11	14	17	25.0	Positive	Yes
HE668478	12624	110	T	C	17	12	21	22.2	Not attempted	No
HE668478	12641	110	T	C	16	11	18	21.5	Not attempted	No
HE670855	10858	37	A	G	25	11	30.5	32.7	Positive	Yes
HE668834	189330	1	G	A	26	23	58	35.6	Positive	Yes
HE671384	187868	4	T	A	29	17	40.5	30.8	Positive	Yes
HE672075	836004	118	G	A	4	12	17	15.6	Positive	Yes
HE669561	219246	110	C	G	21	13	18	19.3	Sequence unreadable	No
HE671439	264572	110	C	T	45	11	28.5	30.2	Negative	No
HE669870	4463	33	G	C	24	13	53	34.2	Positive	Yes
HE671028	12549	110	CA	C	16	11	60.5	25.7	Sequence unreadable	No
HE671010	9591	4	T	A	21	21	24.5	22.0	Sequence unreadable	Yes

Figure S1. Screenshot from IGV showing variants called in contig HE668478 containing two candidate mutations (locations 12624, 12641) that are likely to be false positives. Grey horizontal bars represent sequencing reads, and nucleotides that are different from the reference sequence (shown at the bottom) are indicated.

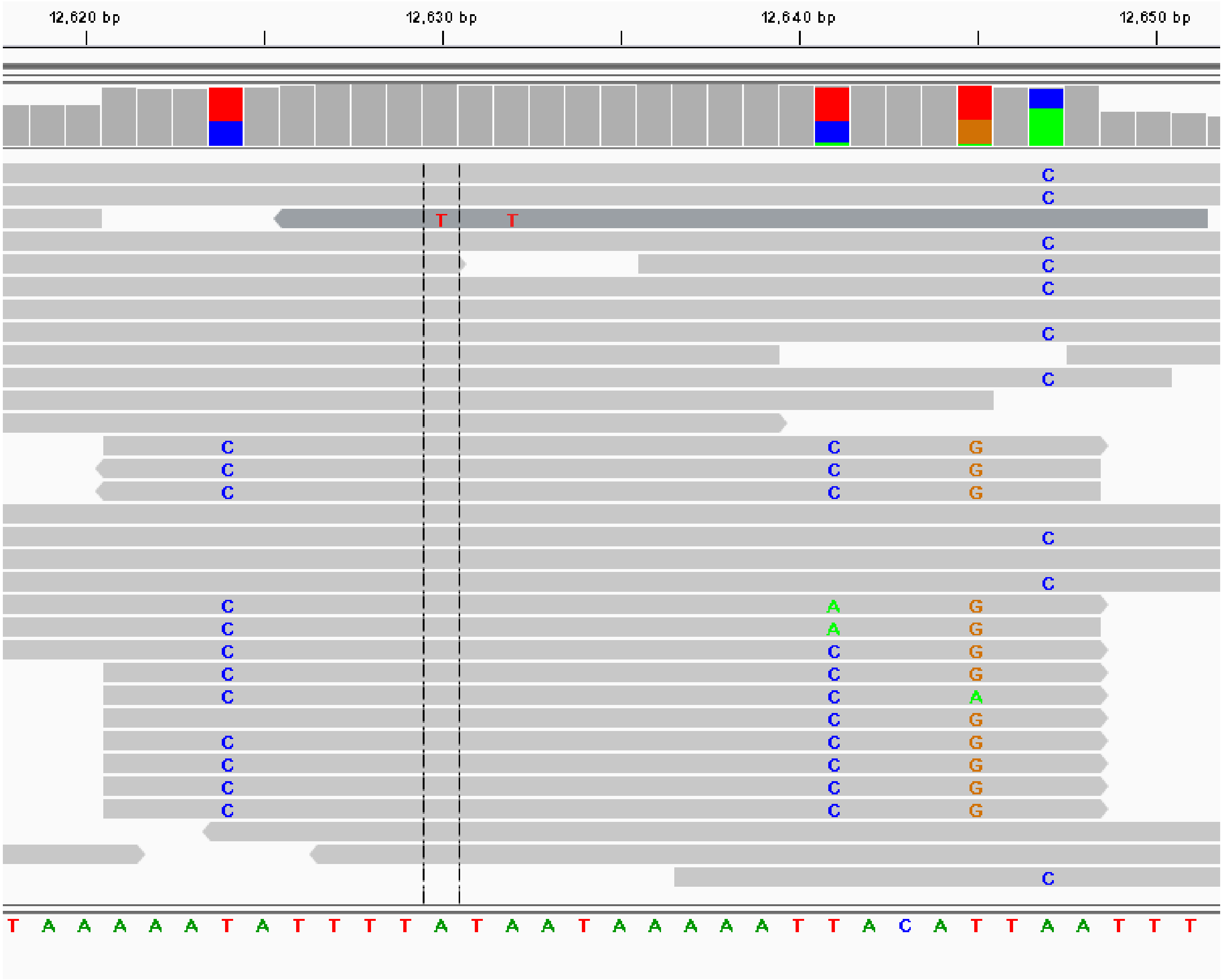

Figure S2. Screenshot from IGV showing a variant called in contig HE671028 (indicated by vertical dashed lines) in individual 110 (upper panel) containing a single nucleotide (A) deletion candidate mutation that is likely to be a false positive, due to the presence of SNPs in complete association with it that are absent from other individuals. Reads from individual 2 (lower panel) are shown for comparison.

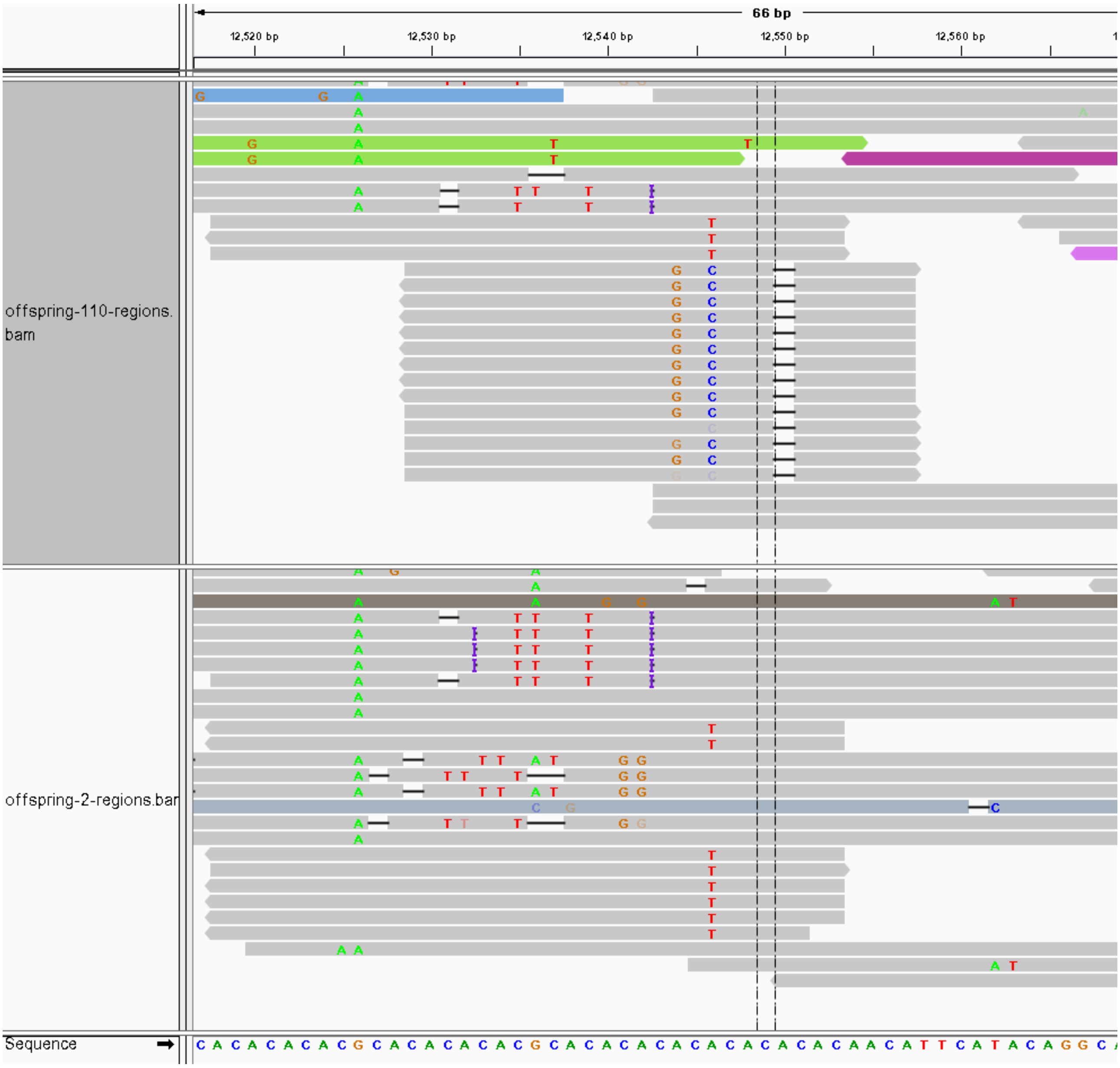

Figure S3. Screenshot from IGV showing a variant called in contig HE669561 (indicated by vertical dashed lines) in individual 110 (upper panel) containing a C->G candidate mutation that is likely to be a false positive, due to the presence of a SNP in association with it that are absent from other individuals. Reads from individual 4 (lower panel) are shown for comparison.

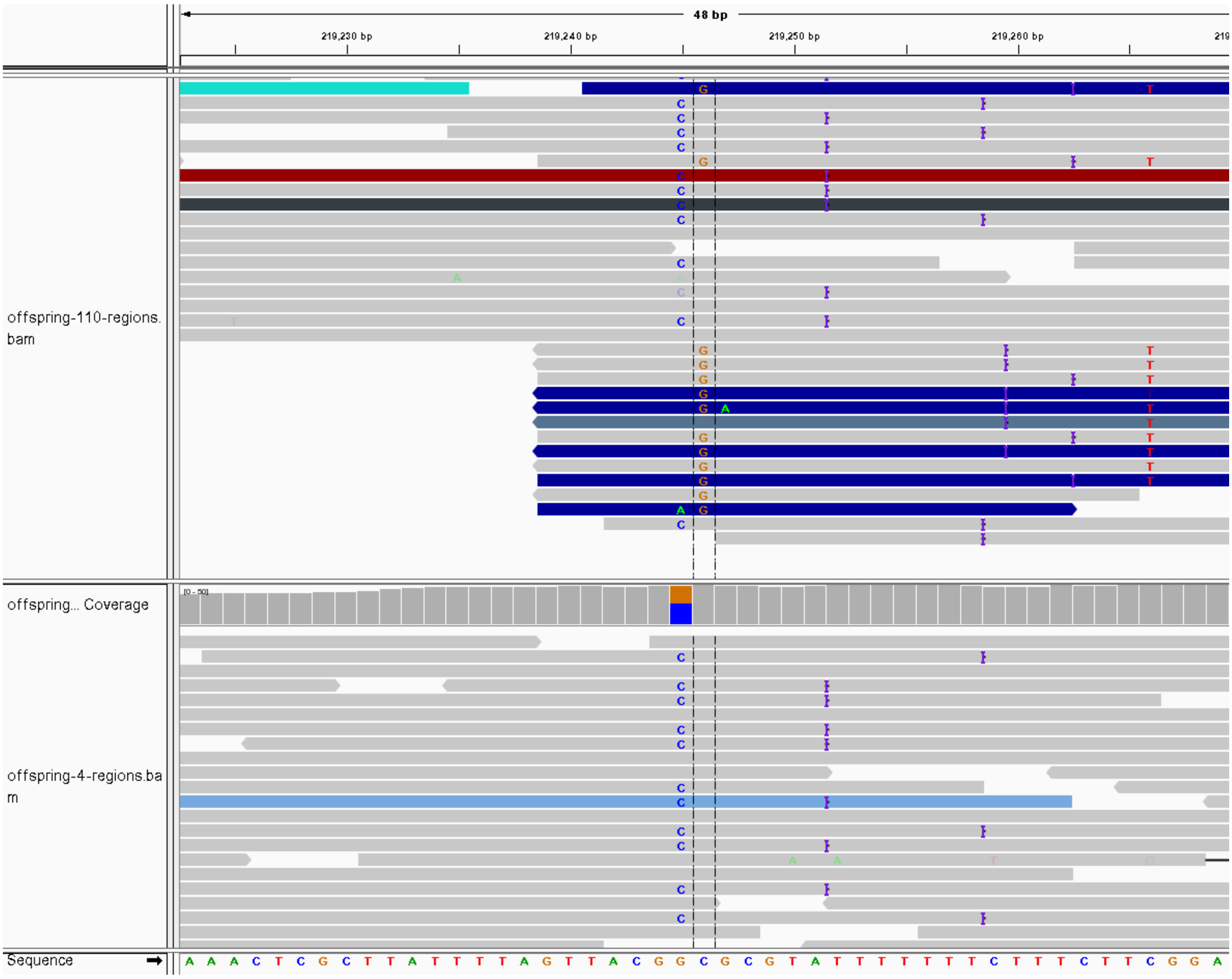

Figure S4. Screenshot from IGV showing a variant called in contig HE671010 (indicated by vertical dashed lines) in individual 4 (upper panel), which is likely to be a genuine mutation. Reads from individual 1 (lower panel) are shown for comparison.

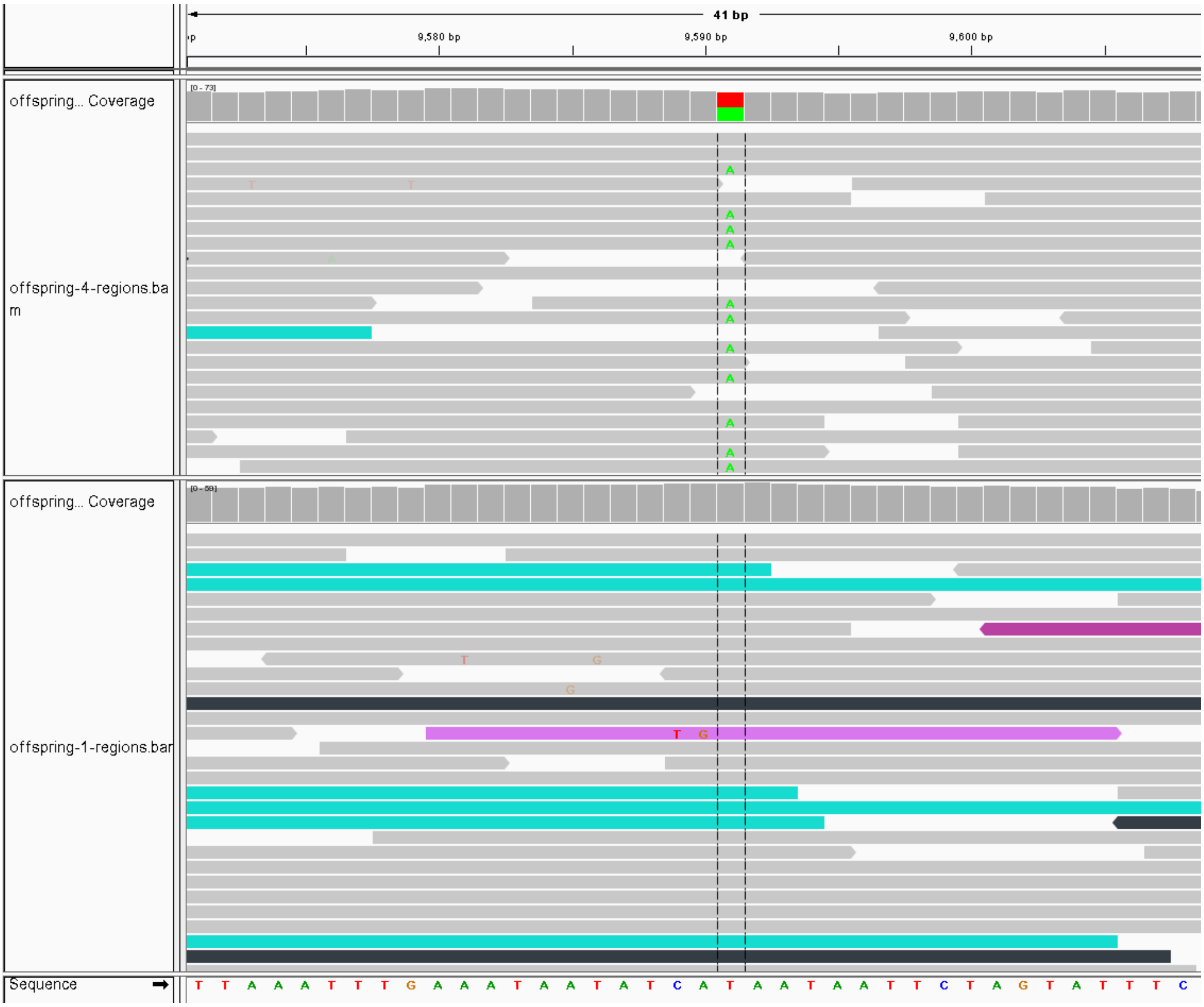

Supplement: Supplementary Data [file supp_msu302_supplementary-211014.pdf]
